# Anticholinergic burden and polypharmacy in patients referred from primary care to tertiary dementia centers in Brazil

**DOI:** 10.1590/1980-5764-DN-2024-0246

**Published:** 2025-08-22

**Authors:** Raphael Machado Castilhos, Carolina Rodrigues Formoso, Wyllians Vendramini Borelli, Elaine Calumby Teixeira, Gabriella Corrêa Dousseau, Márcia Lorena Fagundes Chaves, Sonia Maria Dozzi Brucki

**Affiliations:** 1 Hospital de Clínicas de Porto Alegre, Serviço de Neurologia, Centro de Neurologia Cognitiva e Comportamental, Porto Alegre RS, Brazil.; 2 Universidade Federal do Rio Grande do Sul, Programa de Graduação em Ciências Médicas, Porto Alegre RS, Brazil.; 3 Universidade Federal do Rio Grande do Sul, Faculdade de Medicina, Porto Alegre RS, Brazil.; 4 Universidade Federal do Rio Grande do Sul, Programa de Farmacologia e Terapêutica, Porto Alegre RS, Brazil.; 5 Hospital Santa Marcelina, São Paulo SP, Brazil.; 6 Universidade Federal do Rio Grande do Sul, Departamento de Medicina Interna, Porto Alegre RS, Brazil.

**Keywords:** Polypharmacy, Dementia, Referral and Consultation, Primary Health Care, Polimedicação, Demência, Encaminhamento e Consulta, Atenção Primária à Saúde

## Abstract

Anticholinergic burden (ACB) and polypharmacy are poorly studied in the context of primary care in Brazil.

**Objective:**

To evaluate the ACB and polypharmacy of individuals with suspected dementia referred from primary care to tertiary dementia outpatient clinics in Brazil.

**Methods:**

We performed a cross-sectional study in two tertiary dementia clinics. We included individuals with suspected dementia referred from primary care. Sociodemographic variables, number of drugs, ACB score, disease duration, Mini Mental State Examination (MMSE) were collected in the first evaluation. Final diagnosis received was also collected.

**Results:**

A total of 921 individuals were included, with a median (IQR) age of 72 [64–78] years, 57.8% (532) women, 4 [2–7] years of formal education and 15 [10–20] points in MMSE. Most patients had a final diagnosis of dementia (66%, 616) and Alzheimer’s disease (21.4%, 197), psychiatric disorders (16%, 147) and multifactorial dementia (14.8%, 136) were the most common diagnoses. Most individuals (68.1%, 627) were using at least one medication with anticholinergic effect, and in 44.6% (411) there was polypharmacy. ACB total score correlated with MMSE (rho=-0.13) and with total number of medications (rho=0.52). In multivariate regression, ACB score ≥1 was associated with MMSE and polypharmacy.

**Conclusion:**

Most individuals referred from primary care in Brazil were using at least one medication with anticholinergic effect and this was correlated with cognitive severity. Educational measures for primary care physicians, focusing on the management of pharmacological treatment, are essential to reduce the anticholinergic load in this context.

## RESUMO

A carga anticolinérgica (CAC) e a polifarmácia são pouco estudadas no contexto da atenção primária no Brasil.

**Objetivo:**

Avaliar a CAC e a polifarmácia de indivíduos com suspeita de demência encaminhados da atenção primária para clínicas ambulatoriais de demência terciárias no Brasil.

**Métodos:**

Foi realizado um estudo transversal em dois ambulatórios terciários de demência. Foram incluídos indivíduos com suspeita de demência encaminhados da atenção primária. Variáveis ​​sociodemográficas, número de medicamentos, escore CAC, duração da doença, Mini Exame do Estado Mental (MEEM) foram coletadas na primeira avaliação. O diagnóstico final recebido também foi registrado.

**Resultados:**

Um total de 921 indivíduos foram incluídos, com idade mediana (IIQ) de 72 [64–78] anos, 57,8% (532) mulheres, 4 [2–7] anos de educação formal e 15 [10–20] pontos no MEEM. A maioria dos pacientes recebeu diagnóstico final de demência (66%, 616), sendo a doença de Alzheimer (21,4%, 197), transtornos psiquiátricos (16%, 147) e demência multifatorial (14,8%, 136) os diagnósticos mais comuns. A maior parte dos indivíduos (68,1%, 627) utilizava ao menos um medicamento com efeito anticolinérgico, e em 44,6% (411) foi observada polifarmácia. O escore total da CAC correlacionou-se com o MMSE (rho=- 0,13) e com o número total de medicamentos (rho=0,52). Na regressão multivariada, o escore CAC≥1 esteve associado ao MMSE e à polifarmácia.

**Conclusão:**

A maioria dos indivíduos encaminhados da atenção primária no Brasil utilizava ao menos um medicamento com efeito anticolinérgico, e esse uso esteve correlacionado com a gravidade cognitiva. Medidas educativas voltadas a médicos de atenção primária, com foco no gerenciamento do tratamento farmacológico, são essenciais para reduzir a carga anticolinérgica nesse contexto.

## INTRODUCTION

Dementia is a public health problem, with an estimated 50 million people currently affected worldwide^1^. As the population ages, prevalence is expected to rise, placing a considerable burden on healthcare systems, especially in low- and middle-income countries^2^. Although non-pharmacological approaches are available for most clinical manifestations of dementia, medication use to control symptoms is common. Older adults, particularly patients with dementia, are vulnerable to side effects from commonly used drugs^3^.

Anticholinergic burden (ACB) refers to the cumulative effect of one or more drugs with anticholinergic properties. Studies have shown that high ACB is associated with poorer cognitive outcomes, increased risk of falls, and higher mortality rates in older adults^4^. Medications with anticholinergic effects may cause various neuropsychiatric and systemic side effects, such as constipation, urinary retention, confusion, and excessive sedation^5^. Despite these risks, anticholinergic medications are frequently prescribed to patients with dementia for indications including urinary incontinence, depression, agitation, and sleep disturbances^6^.

Polypharmacy, defined as the concurrent use of multiple medications, is particularly prevalent among patients with dementia^5^. This population is often prescribed various drugs to manage not only cognitive and behavioral symptoms of dementia but also comorbid conditions such as hypertension, diabetes, and cardiovascular diseases. While polypharmacy can be beneficial in addressing these multifaceted health issues, it also increases the risk of adverse drug reactions, drug-drug interactions, and medication non-adherence^7^.

This study aimed to investigate ACB and polypharmacy in patients referred from primary care to two Brazilian tertiary dementia centers. By examining the medication profiles of these patients, the prevalence and patterns of anticholinergic medication use will be identified, and overall ACB assessed. Understanding these factors is crucial for optimizing pharmacotherapy, improving patient outcomes, and reducing the risks associated with polypharmacy and high anticholinergic burden in dementia care.

## METHODS

A retrospective analysis was conducted on all referrals to two large tertiary memory clinics in Brazil: Hospital de Clínicas de Porto Alegre (HCPA) (Porto Alegre, Southern Brazil) and Hospital Santa Marcelina (HSM) (São Paulo, Southeast Brazil) from June 2014 to June 2022. Individuals included in this study were referred by a general practitioner or family physician from the primary care setting of the public health system (*Sistema Unico de Saúde* — SUS).

Patients underwent a routine evaluation comprising a semi-structured interview, including cognitive screening, depression assessment, neurological examination, and laboratory/neuroimaging exams to exclude potentially reversible causes of dementia. Subsequently, patients were classified into dementia syndromes according to international diagnostic criteria as follows: Alzheimer disease (AD) dementia^8^, mild cognitive impairment (MCI)^9^, vascular dementia (VD)^10^, Frontotemporal dementia (FTD)^11^, Lewy bodies dementia^12^, normal pressure hydrocephalus (NPH)^13^. Individuals with normal cognition with or without cognitive complaints were classified as “cognitively unimpaired” (CU). When more than one likely etiology was present, the classification ”multifactorial” was used. Individuals with cognitive impairment or complaints secondary to psychiatric disorders were classified separately. Conditions such as Lewy body dementia, primary progressive aphasia, behavioral frontotemporal dementia, and alcohol dementia were uncommon (≤10 individuals) among primary care referrals; these were grouped as “other dementia” (Suppl. Table 1). Individuals with advanced stages of dementia, with indistinguishable clinical characteristics at disease onset, were classified as “unspecified dementia.”

Several variables were collected during the initial evaluation at each center: age, gender, disease duration, years of formal education, number of medications in use, Mini Mental State Examination (MMSE)^14^. ACB was calculated using the Brazilian anticholinergic activity drug scale^15^. This scale classified each medication according to its anticholinergic activity from 0 (no anticholinergic activity) to 3 (high anticholinergic activity). The total ACB score for each individual was defined as the sum of the scores for each medication in use. As no consensus exists to define polypharmacy, the most common definition of more than five medications was applied^16^. For this analysis, only individuals with a final diagnosis established were included; thus, this variable was collected retrospectively from medical records rather than during the first evaluation.

Frequencies and categorical variables were compared using χ^2^ tests. Continuous variables were presented as median and interquartile range (IQR), as normality analysis (Shapiro-Wilk test and histogram visualization) indicated non-normal distribution. ACB was analyzed both as a quantitative variable and dichotomized as 0 or ≥1 medication. Correlations were assessed with the Spearman coefficient. A multivariate logistic regression model was conducted using age, gender, education, disease duration, diagnosis, total number of medications, polypharmacy, and MMSE as predictors of ACB score and polypharmacy. Additionally, multiple linear regression was performed with MMSE as the outcome and ACB and polypharmacy as independent variables, controlling for gender, age, center of origin, education, disease duration, and final diagnosis. All analyses were conducted using R software version 4.2.2^17^.

### Ethics approval statement

This study was approved by the Institutional Review Board of HCPA (Office for Human Research Protections: #6643121). Informed consent was obtained from all individual participants included in the study.

## RESULTS

A total of 921 referrals were included, with 56.3% (519) from HSM and 43.6% (402) from HCPA. The median (IQR) age of patients was 72 [64–78] years, and 57.8% (532) were women with a median (IQR) of 4 [2–7] years of formal education. Differences in sociodemographic variables between the two centers are detailed in Table 1. Individuals with dementia had a median (IQR) disease duration of 2 [1–4] years, with no differences between etiologies, and a median (IQR) MMSE score of 15 [10-20] points. Most patients received a final diagnosis of dementia (66%, 616), with AD (21.4%, 197), psychiatric disorders (16%, 147), and multifactorial dementia (14.8%, 136) being the most common single diagnoses (Table 2, Suppl. Table 2, Suppl. Table 3).

**Table 1:** Sociodemographic and clinical characteristics of the sample, divided dementia diagnosis.

	Dementia	Not dementia	Total	p-value
n (%)	608 (66)	313 (34)	921	-
Age median (IQR)	74 (66–79)	69 (61–75)	72 (64–78)	<0.001 ^a^
Female n (%)	320 (52.6)	212 (67.7)	532 (57.8)	<0.001 ^b^
Education (years) median (IQR)	4 (1–6)	4 (2–8)	4 (2–7)	0.008 ^a^
Disease duration median (IQR)	2 (1–4)	2 (1–5)	2 (1–4)	0.360 ^a^
MMSE median (IQR)	15 (10–20)	23 (20–26)	18 (13–23)	<0.001 ^a^
ACB total score median (IQR)	1 (0–3)	1 (0–3)	1 (0–3)	0.203 ^a^
ACB score ≥1 n (%)	426 (70.1)	201 (64.2)	627 (68.1)	0.084 ^b^
Number of medications median (IQR)	4 (2–6)	4 (2–6)	4 (2–6)	0.525 ^a^
Polypharmacy n (%)	272 (44.7)	139 (44.4)	411 (44.6)	0.980 ^b^

Abbreviations: IQR, interquartile range; MMSE, Mini Mental State Examination; ACB, Anticholinergic Burden score.

Notes: ^a^Mann-Whitney;

^b^χ^2^.

**Table 2:** Anticholinergic burden score, polypharmacy, and total number of medications according to the final diagnosis.

	ACB total score^a^ median (IQR)	ACB score ≥1^b^ n (%)	Total number of medications^c^ median (IQR)	Polypharmacy^d^ n (%)
**Whole sample (n=921)**	1 (0–3)	627 (68.1)	4 (2-6)	411 (44.6)
**Individuals with dementia**				
Total (n=616)	1 (0–3)	426 (70.1)	4 (2–6)	272 (44.7)
Alzheimer’s disease (n=197)	1 (0–2)	132 (67)	4 (2–5)	66 (33.5)
Multifactorial dementia (n=136)	2 (1–3)	105 (77.2)	5 (3–7)	78 (57.2)
Vascular dementia (n=112)	1 (0–3)	82 (73.2)	6 (4–8)	71 (63.4)
Dementia unspecified (n=66)	1 (0–3)	42 (63.6)	4 (1.25–6.75)	28 (42.4)
Other diagnosis (n=39) ^e^	1 (0–3)	22 (56.4)	2 (0–6)	14 (35.9)
Traumatic Brain Injury (n=19)	1 (0-2)	13 (68.4)	1 (1-3)	3 (15.8)
Normal pressure hydrocephalus (n=13)	1 (0–1)	8 (61.5)	4 (2–6)	6 (46.2)
Alcohol (n=12)	2 (1–3.25)	10 (83.3)	3 (2.75–4)	1 (8.3)
Synucleinopathy (n=12)	2.5 (1–4)	11 (91.7)	4 (2.75–6.25)	5 (41.7)
Frontotemporal dementia (n=10)	0.5 (0–1.75)	5 (50)	2.5 (0–4.75)	3 (30)
**Individuals without dementia**				
Total (n=305)	1 (0–3)	201 (64.2)	4 (2–6)	139 (44.4)
Psychiatric disorder (n=147)	1 (0–3)	104 (70.7)	5 (2–7)	75 (51)
Mild cognitive impairment (n=116)	1 (0–2)	71 (61.2)	4 (2–6)	46 (39.7)
Cognitively unimpaired (n=42)	1 (0–2)	22 (52.4)	4 (2–5)	15 (35.7)

Abbreviations: ACB, Anticholinergic Burden.

Notes: ^a^p=0.002, Kruskal-Wallis;

^b^p=0.023, χ^2^;

^c^p<0.0001, Kruskal-Wallis;

^d^p<0.001, χ^2^;

^e^A list of diagnosis included in this category are detailed in the Supplementary Material.

**Figure 1:**
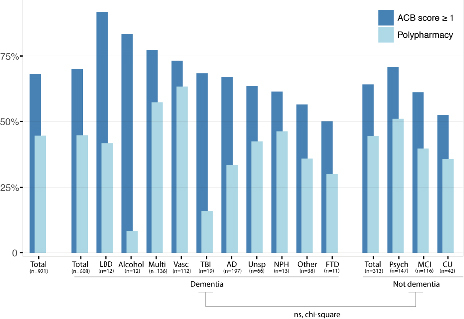
Anticholinergic burden and polypharmacy among the final diagnosis.

Abbreviations: ACB, anticholinergic burden; LBD, Lewy Body dementia; Multi, Multifactorial dementia; Vasc, Vascular dementia; TBI, Traumatic Brain Injury; AD, Alzheimer disease; Unsp, Unspecified dementia; NPH, Normal pressure hydrocephalus; Other, Other dementia causes; FTD, Frontotemporal dementia; Psych, Psychiatric disorders; MCI, Mild cognitive impairment; CU, cognitive unimpaired.

Most patients (68.1%, 627) were using at least one medication with anticholinergic effects, and 44.6% (411) met the criteria for polypharmacy. The median (IQR) total ACB total score was 1 [0-3], with no significant differences in total ACB score or percentage of polypharmacy between individuals with and without dementia (Figure 1, Table 2). The frequency of ACB≥1 differed by dementia etiology. Patients with Lewy body dementia (LBD) (91.7%, 11), alcohol-related dementia (83.3%, 10), and multifactorial dementia (77.2%, 105) more frequently had ACB≥1. Polypharmacy was more common in vascular dementia (63.4%, 71) than in other diagnoses (Figure 1 and Table 2).

Most prescriptions for medications with anticholinergic effects were for those classified as ACB score=1 (76.7%, 419/546). The most commonly prescribed classes were antidepressants (35.1%), antihypertensives (18.1%), and antipsychotics (17.4%). In this sample, 19.3% were using anti-dementia medications, mainly donepezil and memantine (Suppl. Table 4).

**Figure 2:**
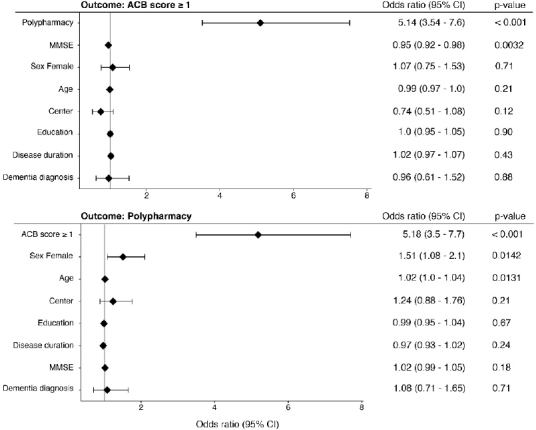
Forest plots of multivariate logistic regression of anticholinergic burden score≥1 and polypharmacy as the dependent variable.

Abbreviations: ACB, Anticholinergic burden; 95%CI, 95% confidence interval; MMSE, Mini Mental State Examination.

The total ACB score did not correlate with age and education but showed weak correlations with disease duration (rho=0.07) and MMSE (rho=-0.13), and a moderate correlation with the total number of medications (rho=0.52). The total number of medications was correlated only with age (rho=0.10) and the total ACB score (rho=0.52). The frequencies of ACB total score≥1 and polypharmacy were associated: 56.8% (356) of those with ACB≥1 having polypharmacy, compared to only 18.7% (55) of those with ACB=0 (χ^2^, p<0.001) (Table 3). In multivariate logistic regression, ACB≥1 was associated with MMSE and polypharmacy. When polypharmacy was used as the dependent variable in the logistic regression, ACB≥1, age, and female gender were associated with the outcome (Figure 2). In multiple linear regression, controlling for sociodemographic and clinical variables, neither ACB nor polypharmacy was associated with MMSE.

**Table 3:** Sociodemographic and clinical characteristics of individuals with Anticholinergic burden score≥1 and polypharmacy.

	ACB≥1 (n=627)	ACB=0 (n=294)	Polypharmacy (≥5 drugs) (n=411)	No polypharmacy (<5 drugs) (n=510)
Age median (IQR)	72 [65, 78]	70.5 [63, 78]	73 [66, 78]	71 [63, 78]
Female n (%)	373 (59.5)	159 (54.1)	261 (63.5) ^b^	271 (53.1)
Education (years) median (IQR)	4 [2, 6]	4[1.75, 7]	4 [2, 6]	4 [2, 8]
Disease duration median (IQR)	2 [1, 5]	2 [1, 4]	2 [1, 5]	2 [1, 4]
MMSE median (IQR)	17 [12, 22] ^a^	20 [15, 23]	18 [12, 22]	18 [13, 23]
Dementia n (%)	426 (67.9)	182 (61.9)	272 (66.2)	336 (65.9)
ACB total score median (IQR)	2 [1, 3]	-	2 [1, 4] ^a^	1 [0, 2]
ACB score≥1 n (%)	-	-	356 (86.6) ^a^	271 (53.1)
N of medications median (IQR)	5 [3, 7] ^a^	2 [0, 4]	7 [5, 8] ^a^	2 [1, 3]
Polypharmacy n (%)	356 (56.8) ^a^	55 (18.7)	-	-

Abbreviations: ACB, Anticholinergic burden; IQR, interquartile range; MMSE, Mini Mental State Examination.

Notes: ^a^p<0.001;

^b^p=0.002.

## DISCUSSION

We observed a high ACB and polypharmacy among referrals to two Brazilian tertiary care dementia centers. These centers receive a particular type of referral, as most patients are sent directly from primary care without prior evaluation by a secondary care physician or undergoing any preliminary screening. Consequently, patients arriving at our memory clinics often experience long delays before evaluation, as previously demonstrated^18^. This characteristic of our sample is reflected in the fact that approximately 20% of individuals did not have dementia, and most patients were in advanced stages of the disease, as reported in a previous study by our group^19^. Despite this delay, we found that two-thirds of patients were using at least one medication with anticholinergic properties, and nearly half were taking more than five medications (the definition of polypharmacy used in this study). These proportions are higher than those observed in an Italian survey (~25%) of older adults with cognitive complaints in primary care^20^ and an Irish study (~33%) involving individuals with dementia/MCI in primary care^21^. Thes findings may reflect a tendency toward liberal prescribing practices in our primary care context. Another possible explanation is that patients referred to our clinics may have more pronounced neuropsychiatric symptoms. It is well known that primary care physicians often encounter difficulties in managing such cases, which is a major reason for referral^22^. Moreover, implementing non-pharmacological treatment in primary care is challenging due to a lack of training and confidence, leading to a preference for pharmacological interventions^23^. This may explain the high frequency of psychotropic medication use in our sample.

In our sample, ACB and polypharmacy were associated, which is expected, as the likelihood of prescribing a drug with anticholinergic properties increases with the number of medications used^24^. However, the association with cognitive impairment severity (MMSE) was only associated (despite being weak) with ACB, suggesting that anticholinergic properties, more than the number of drugs used, are driving the worsening of cognitive impairment, as suggested by the study with the UK Biobank^25^. In our study, the most frequent dementia etiologies with at least one drug with anticholinergic effect were LBD, alcohol-related, multifactorial, and vascular dementia. This could be due in part to the fact that these etiologies are very often associated with neuropsychiatric manifestations and the risk of prescription of medications with anticholinergic effects^26-28^. Specifically in vascular dementia, the higher frequency of comorbidities seen in these patients could also explain the findings. Additionally, the high frequency of polypharmacy and anticholinergic burden in patients with psychiatric illness may be due to the fact that these patients frequently use antidepressants and antipsychotics with some anticholinergic effect.

The use of anticholinergics is associated with several consequences for older adult patients, especially those with dementia, including all-cause mortality, cognitive decline, and poor quality of life^29,30^. Although patients are using various classes of psychotropics, many medications have hidden anticholinergic effects, such as antihypertensives and diuretics^5^. In our sample, about 20% used antihypertensives with some anticholinergic action, mainly captopril, atenolol, and metoprolol. In addition, a recent meta-analysis found some evidence that CU adults present a higher risk of cognitive decline due to anticholinergic medication^31^ and that there is a dose-dependent relationship between ACB and dementia risk^32^. In addition, ACB is associated with many prognostic factors in older adults, and active reduction of these medications is beneficial for patients^33^.

This study presents some limitations. Firstly, this is a retrospective study and therefore presents biases related to this type of design, such as data collection that was not previously planned. Moreover, the anticholinergic burden may vary according to the scale used^34^, so our results could differ if other measures were applied. In any case, the scale we used^15^ was adapted to the medications available in Brazil, so we consider these results valid for our context. Additionally, our sample is not representative of the older adult population in primary care and is not comparable to previous studies that evaluated inappropriate medication use in the primary care context^35^. Further, as our study spans several years (including the pandemic period), it is likely that different referral routines were in place, which may have influenced our results. Unfortunately, we did not have further information about the reasons for referral, other than the general suspicion of dementia. Finally, the instrument we used does not account for the dosage of each drug, so patients with the same score may be using different doses and thus have different anticholinergic burden. Despite these limitations, our findings are original, as our analysis is the first to evaluate ACB and polypharmacy in patients with suspected dementia referred from primary care in Brazil. With these results, we hope to help improve the quality of pharmacological management of patients with dementia in our country.

## Data Availability

The data are available at https://www.demneuropsy.com.br/wp-content/uploads/2025/04/DN-2024.0246-Supplementary-Material.docx.
